# The dynamics of HIV trends of transmission in the Romanian cohort

**DOI:** 10.1186/1471-2334-14-S4-O3

**Published:** 2014-05-29

**Authors:** Mariana Mărdărescu, Adrian Streinu-Cercel, Dan Oțelea, Marieta Iancu, Sanda Vintilă, Iosif Ionel

**Affiliations:** 1 National Institute for Infectious Diseases "Prof. Dr. Matei Balş", Bucharest, Romania; 2 Carol Davila University of Medicine and Pharmacy, Bucharest, Romania; 3 National Institute of Public Health, Romania

## 

Since the early 1990s Romania has made important progresses in the HIV/AIDS area, also recognised by the international community. These steps forward concern treatment and care for people living with HIV/AIDS (PLWHA) and prevention of HIV transmission among young people and vulnerable groups. However, the global economic crisis has generated a behavioural change especially among the young population, with an increase of incidence at 2.54/100,000 (31 December 2013).

At the end of 2013 we performed the annual statistical evaluation of the HIV epidemic. The National Database registered 19,261 cases of HIV and AIDS (cumulative total 1985-2013), of which 12,273 were PLWHA. Most cases (65%) were diagnosed when they were children (<14) at the beginning of the 1990s and since then have experienced multiple ART regimens and reached a fertile age.

Most of the new registered cases (797) represent young PLWHA (20-24, 25-29) who suffered changes in their behavioural patterns. Hence, the former intravenous drug users (IDUs) shifted to daily use of new drugs (ethnobotanicals) while young women acquired HIV through unprotected sex and i.v. drug use.

Another area of concern was mother to child transmission of HIV that we assessed in the context of prenatal and postnatal cares.

Since 2011, the national response to HIV has weakened as a consequence of the economy. Hence, the new IDU-HIV cases boosted from 14 cases (3%) in 2010 to 233 (29.23%) in 2013. At the same time we detected a growth in the share of children born from women using drugs, including women living with HIV/AIDS (WLHA). For the same category we identified 76.83% HCV co-infections, 11% HBV-HCV co-infections. The assessment evinced that late presenters account for more than 50% from the new cases, with CD4<350 cells/cmm. In addition, 32% of patients in treatment have low CD4 counts <350 cells/cmm (end 2013).

The rising number of pregnant women addicted to drugs is directly proportional to the expanding figures of drugs and new drugs users. Cares provided to newborns from mothers who use i.v drugs and new drugs, perinatally exposed to HIV are usually associated with hepatitis B, C and with syphilis.

Romania needs an upgrade of its national HIV/AIDS policies: broaden access to treatment and integrated services, using ART as prevention measure to avoid HIV transmission among the general population and a strengthened partnership between the medical networks, in order to respond to the emergent HIV trends.

